# Energy Expenditure Prediction from Accelerometry Data Using Long Short-Term Memory Recurrent Neural Networks

**DOI:** 10.3390/s24082520

**Published:** 2024-04-14

**Authors:** Martin Vibæk, Abdolrahman Peimankar, Uffe Kock Wiil, Daniel Arvidsson, Jan Christian Brønd

**Affiliations:** 1 SDU Health Informatics and Technology, The Maersk Mc-Kinney Moller Institute, University of Southern Denmark, 5230 Odense, Denmarkabpe@mmmi.sdu.dk (A.P.); 2 Center for Health and Performance, Department of Food and Nutrition, and Sport Science, Faculty of Education, University of Gothenburg, 405 30 Gothenburg, Sweden; daniel.arvidsson@gu.se; 3 Department of Sport Science and Clinical Biomechanics, University of Southern Denmark, 5230 Odense, Denmark; jbrond@health.sdu.dk

**Keywords:** accelerometry, deep learning, LSTM, children, energy expenditure

## Abstract

The accurate estimation of energy expenditure from simple objective accelerometry measurements provides a valuable method for investigating the effect of physical activity (PA) interventions or population surveillance. Methods have been evaluated previously, but none utilize the temporal aspects of the accelerometry data. In this study, we investigated the energy expenditure prediction from acceleration measured at the subjects’ hip, wrist, thigh, and back using recurrent neural networks utilizing temporal elements of the data. The acceleration was measured in children (N = 33) performing a standardized activity protocol in their natural environment. The energy expenditure was modelled using Multiple Linear Regression (MLR), stacked long short-term memory (LSTM) networks, and combined convolutional neural networks (CNN) and LSTM. The correlation and mean absolute percentage error (MAPE) were 0.76 and 19.9% for the MLR, 0.882 and 0.879 and 14.22% for the LSTM, and, with the combined LSTM-CNN, the best performance of 0.883 and 13.9% was achieved. The prediction error for vigorous intensities was significantly different (*p* < 0.01) from those of the other intensity domains: sedentary, light, and moderate. Utilizing the temporal elements of movement significantly improves energy expenditure prediction accuracy compared to other conventional approaches, but the prediction error for vigorous intensities requires further investigation.

## 1. Introduction

Today, children and adolescents spend more and more time being sedentary [1,2] and there is an unfavorable association between the amount of time that they spend being sedentary and their physical and mental health [3]. Physical inactivity is suggested as one of the main causes of public chronic diseases such as obesity, diabetes, coronary heart disease, and depression [4]. Having simple and accurate methods for estimating children’s physical activity (PA) from the aspects of their energy expenditure (EE) provides researchers and policymakers with important tools to monitor public health, evaluate the effectiveness of proposed interventions, or further investigate the association between physical behavior and health.

Physical activity is defined as any bodily movement produced by skeletal muscle that results in EE [5], and different technologies are available for assessing the PA from the aspect of EE. Not all methods can be used in a natural environment and record for multiple days, which is required to provide a robust estimation of a subject’s general PA [6]. Activity monitors which measure acceleration are a simple technology which can be used in a natural environment and provides a sufficient recording duration. The objective nature of this methodology is not affected by measurement bias like self-report assessments are [7]. Over the last decade, the activity monitor device size has been reduced and additional sensors like light, temperature, or gyroscope sensors have been added. Even though accelerometry has been shown to be robust, it is known that the intensity estimated with activities like basketball, soccer, stair walking, and biking is associated with substantial measurement errors [6]. The known measurement errors have inspired the development of multiple prediction algorithms which provide a more accurate estimate of intensity and, thus, EE [8,9,10,11,12,13,14,15,16,17,18,19,20]. Moreover, obtaining high accuracy EE predictions in children is especially challenging due the intermittent and sporadic nature of their movement behavior [21], as well as their smaller size, lower body weight, VO_2MAX_ (maximal oxygen consumption), and higher resting basic metabolic rates [22,23,24]. If the accuracy of estimating the intensity of sporadic and intermittent PA is poor, the determined prevalence of meeting the World Health Organization recommendations of 60 min/day for children will remain underestimated. This can have important implications for how policy makers prioritize PA initiatives.

**Figure 1:**
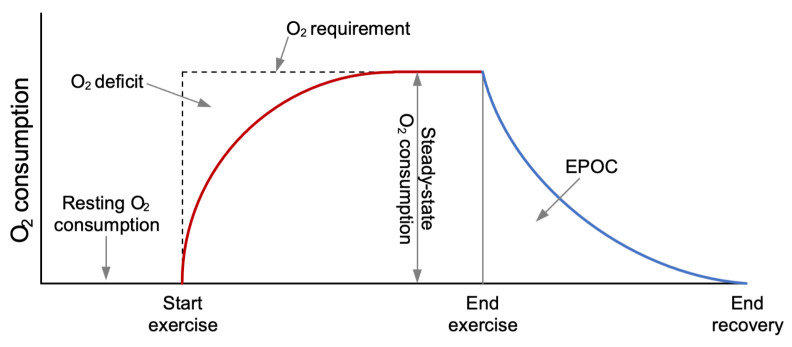
Oxygen uptake and, thus, energy expenditure during an activity/exercise.

The coverage of energy by the aerobic system during an activity is presented in Figure 1, and plays an important role in activities with a duration longer than 6–10 s. In the first few seconds, the energy requirements are mainly covered by the immediate storage, which is reestablished by ending muscle contractions. The reestablishment of the immediate storage and the removal of lactate are covered by the aerobic system with an excess post-exercise oxygen consumption (EPOC), which consists of a rapid and a prolonged component [25]. The amount of energy consumed during the EPOC period is the same as the energy covered both by the immediate storage and the glycolytic systems at the start of the activity. Therefore, it is called oxygen deficit. It has been shown that the EE of earlier activities affects the present EE, which is caused by EPOC [25,26]. Consequently, if the time between two bouts of movement is long (>10–20 s), the EE will follow the movement pattern to some extent and EPOC will have enough time to reestablish. However, if the time between two bouts of fast movement is short (<10 s), the EE during slow movement or while not moving will be at a similar level as the EE during fast movement [25,26]. The O_2_ deficit is lower with children as compared to adults due to the immaturity of the anaerobic glycolytic system in children. Children’s movement behavior is sporadic, and they often engage in intermittent activities like playground activities, basketball, or soccer, where their behavior alternates between bouts of fast movement like running and bouts of slower movement and even static postures such as walking and standing still, respectively. Thus, this suggests that previous movements and activities are important elements which should be utilized to obtain an accurate prediction of the EE from acceleration.

Although previous studies investigating EE prediction from acceleration have obtained reasonable accuracy [8,9,10,11,12,13,14,15,16,17,18,19,20], there is still potential for accuracy improvements, and, currently, no study has utilized the temporal elements of movement. Therefore, the aim of the present study is to investigate metabolic EE prediction from acceleration by children by utilizing the temporal elements of movement.

## 2. Materials and Methods

### 2.1. Data

A total of 37 children were enrolled from a local school in Odense municipality of Denmark for the data collection. The children were recruited and informed by word of mouth and email through the school office about the study. The data collection was approved by the Ethics Committee of the Region of Southern Denmark (S-20140068) and was carried out in accordance with the Danish Data Protection Agency. All included participants and/or their legal guardian(s) gave written informed consent. Moreover, the protocols were carried out in accordance with relevant guidelines and regulations (i.e., Declaration of Helsinki). Due to equipment malfunction, four of the participants did not provide sufficient data and were removed from the study. All participants were able to speak and understand Danish. To simulate free living conditions, the participants were required to follow a structured activity protocol as described in Table 1 [27]. The participants wore four Axivity AX3 (AX3) accelerometers (Axivity Ltd., Newcastle upon Tyne, UK) placed on the non-dominant wrist, hip, low back, and thigh. The AX3 is a small lightweight unit (11 g) which is easily worn at various positions on the body. The device provides an adjustable measurement range (±2, ±4, ±8, or ±16 g), sampling frequency range of 12.5–3200 Hz, and a 12(13)-bit resolution. The memory and battery provide the option to record acceleration for more than 7 days at a 50 Hz sampling frequency. For this study, the devices were initialized to collect accelerometer data using a ±8 range and with a sampling frequency of 50 Hz. The acceleration was subsequently resampled to 30 Hz. A sampling frequency of 30 Hz is sufficient to capture enough detail of the fast accelerations performed by children [28]. Data preprocessing was done in Matlab R2021b Version 9.11 (The Mathworks Inc., Natick, MA, USA) and prediction model training and evaluation were done in Python 3.9. The statistical significance level was set as α = 0.05.

### 2.2. Energy Expenditure and Accelerometry Preprocessing

Energy expenditure was measured as the oxygen consumption, using the Metamax 3X (CORTEX Biophysik GmbH, Leipzig, Germany) portable metabolic analyzer [29]. The Metamax sampling frequency is 0.1 Hz, and the data were filtered using a first-order low-pass Butterworth filter with a cut-off frequency of 0.015 Hz to reduce noise. The *VO*_2_ in mL^−1^ kg^−1^ min^−1^ was adjusted for resting energy expenditure (REE) by subtracting the mean of the *VO*_2_ for the first three sedentary activities (i.e., sitting, sitting playing tablet, and standing playing tablet), as given in Equation (1).

(1) $${VO}_{2\;Adjusted}={VO}_{2}-\bar{{VO}_{2\;Sed}}\;$$

where $\bar{{VO}_{2\;Sed\;}}$ is the mean of the *VO*_2_ for the first three sedentary activities.

As the primary input features to the EE prediction, Mean Amplitude Deviation (MAD) [30] and ActiGraph Intermittent (AGI) [31] PA metrics were generated from the raw acceleration using an epoch length of 10 s. The MAD is calculated as the mean acceleration amplitude, subtracting the mean to account for the gravitational component, as given in Equation (2).

(2) $$MAD=\frac{1}{n}\sum \left|r_{i}-\bar{r}\right|$$

where *n* is the number of samples in the epoch, *r_i_* is the *i*th resultant sample within the epoch, and $\bar{r}$ is the mean resultant value of the epoch.

The AGI metric is an extension of the ActiGraph counts metric [32,33], but reduces the known measurement error of children’s sporadic or intermittent physical activity [27]. The mathematical description of the ActiGraph count is omitted for clarity but is presented in the study by Neishabouri et al. [32]. The measurement error is reduced by mimicking the intensity pattern of non-cyclic intermittent activities as opposed to cyclic activities like walking or running. Mimicking the pattern is done by interpolating the low-intensity periods between activity bouts if the intensities of the activity bouts before and after are above moderate and if they are less than 10 s apart. The interpolation is done using a 1 s epoch and the 10 s threshold is selected to reflect the rapid EPOC component [25]. In addition to the MAD and AGI PA metrics, the inclination angle, height, arm, and leg length are also used as input features in the EE prediction models. The inclination angle is calculated using the same procedure as that used in the Skotte et al. (2014) activity type classification method [34].

### 2.3. Prediction Models and Data Segmentation

Two Recurrent Neural Network (RNN) model types are investigated, a stacked long short-term memory (LSTM) and a convolutional neural network (CNN-LSTM) model, whereas a standard Multiple Linear Regression (MLR) model is used as reference. The stacked LSTM consists of an input layer followed by consecutive LSTM layers. The output layer is a single cell-dense layer for single time-step forecasting. The CNN-LSTM model consists of an input layer, followed by Convolutional 1D layers for feature extraction, then LSTM layers and a single cell-dense layer as the output. The adaptive moment estimation (ADAM) optimizer was used to train the models using the mean squared error (MSE) as the loss function.

The available dataset was restructured to be considered as a time series prediction problem. Multivariate time series were generated using demographic and accelerometry features. As mentioned in the previous section, the MAD or AGI, inclination angle, height, and arm and leg sizes are considered as inputs and *VO*_2_ as the target variable for the training of the models. The accelerometer data were segmented into chunks of 10 data points, which correspond to 100 s segment lengths. The window step size was set to 10% (1 sample), which leads to a higher number of segments for model training. Time series were not used as the input for the MLR model, since the dimensions of the time series training set have the size of (7984,10,5) and MLR models are unable to be fitted on 3D data. The first nine instances of *VO*_2_ were excluded for the MLR model to ensure that the dimensions of the MLR predictions matched the dimensions of the RNN model predictions. This accounts for the 10 instances of the independent variables needed to create a single time series.

We used the accelerometer data collected from 33 participants, of which 19 participants (58%) were used for training, 4 participants (12%) for validation, and the remaining 10 participants (30%) for testing. The data contain multiple measurements of each participant, which invalidates the assumptions of independence between data samples. Therefore, K-fold cross-validation was not used to ensure that all activities were included in model training and evaluation.

### 2.4. LSTM Layer

**Figure 2:**
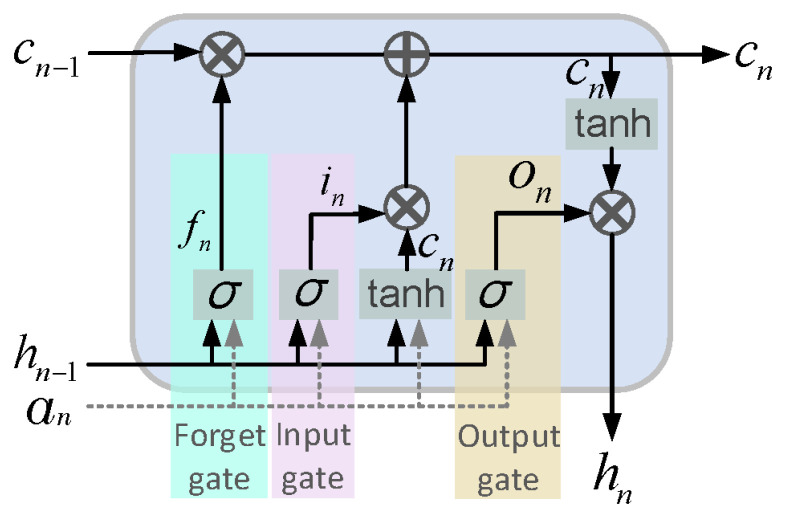
Structure of the LSTM cell.

Recurrent neural networks (RNNs) are designed to handle sequential data, which have been employed for sequential time series applications with temporal dependencies [35]. The main advantage of using RNNs is the capability of these types of networks to use historical data/information for more accurate predictions. Traditional RNNs suffer from learning long-term dependencies in data due to the vanishing gradient problem during the backpropagation [36]. This can be resolved using a special variant of RNNs, which is called long short-term memory (LSTM) and was first introduced by Hochreiter Schmidhuber in 1997 [36]. LSTM addresses the problems of exploding and vanishing gradients, which is usually seen in RNNs. As shown in Figure 2, LSTM consists of three main blocks, the (i) forget gate ($f_{n}$), (ii) input gate ($i_{n}$), and (iii) output gate ($o_{n}$), and continuously updates its memory cell, c_t_. LSTM networks can remove or add information to/from their memory block at each time step in a sequence, which are carefully controlled by the forget gate and the input gate. The input and forget gates employ the same overall structure of a single-layer neural network with a sigmoid activation function, as given in (3) and (4).

(3) $$f_{n}=\sigma \left(b_{f}+u_{f}^{T}x_{n}+w_{f}^{T}h_{n-1}\right),$$

and

(4) $$i_{n}=\sigma \left(b_{i}+u_{i}^{T}x_{n}+w_{i}^{T}h_{n-1}\right),$$

where *x_n_* is the input sequence at time step *n*, and *h*_*n*−1_ is the output of the LSTM at time step *n* − 1. The $u_{i},\;w_{i},\;u_{i},\;\mathrm{and}\;w_{f}$ are actually the weight vectors of the input and forget gates, and *b_f_* and *b_i_* are bias terms, which will be learned over the training phase of the LSTM. The sigmoid activation function (σ) returns a value between 0 and 1, which controls the flow of information passing through each gate. Therefore, the memory cell, $c_{n}$, is updated as:

(5) $$c_{n}=f_{n}c_{n-1}+i_{n}{\tilde{c}}_{n}$$

where

(6) $${\tilde{c}}_{n}=\tanh\left(b_{c}+u_{c}^{T}x_{n}+w_{c}^{T}h_{n-1}\right)$$

Finally, the output of the LSTM cell, *h_n_*, is calculated as:

(7) $$h_{n}=o_{n}\tanh\left(c_{n}\right)$$

where

(8) $$o_{n}=\sigma \left(b_{o}+u_{o}^{T}x_{n}+w_{o}^{T}h_{n-1}\right)$$

The parameters u_o_ and w_o_ are the input and recurrent weight vectors of the output gate, and b_o_ represents the output bias. As given in Equations (4) and (5), in addition to the input and previous output, the output gate is also dependent on the current memory, which provides the LSTM with the ability to efficiently keep or forget the existing state. This helps the network to be capable of remembering features from the early stages of a sequence and hence of capturing long-term dependencies [37,38].

### 2.5. Zero-Padding

**Figure 3:**
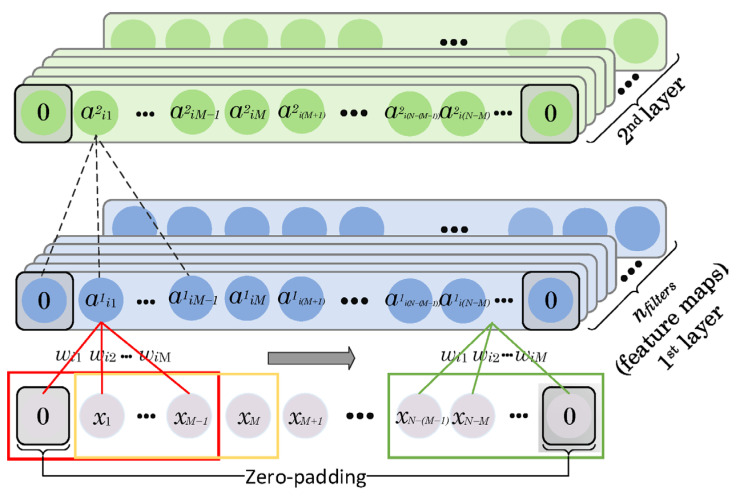
The convolution process. The input time series is traversed by moving a fixed kernel of size $k_{size}=M$. The weights of the kernels $\left(w_{i1},\;w_{i2},\;\cdots ,\;w_{iM}\right)$ are the same for all of the convolution processes. Zero-padding is used to keep the dimension of the time series generated in each layer the same as that of the input signal. For example, if the size of the kernel ($k_{size}$) is an odd number, we pad both ends of the time series with (*M* − 1)/2 zeros, otherwise, the zero-padding is equal to *M*/2 (adapted from [38]).

The convolution process is presented in Figure 3. The input time series is traversed by moving a fixed kernel of size $k_{size}=M$. The weights of the kernels $\left(w_{i1},\;w_{i2},\;\cdots ,\;w_{iM}\right)$ are the same for all the convolution processes. Zero-padding is used to keep the dimension of the time series generated in each layer the same as that of the input signal. For example, if the size of the kernel ($k_{size}$) is an odd number, we pad both ends of the time series with (*M* – 1)/2 zeros, otherwise, the zero-padding is equal to *M*/2.

### 2.6. CNN Layer

CNNs were first proposed by Lecun et al. in 1998 [39]. Unlike fully connected neural networks, CNNs are capable of extracting various spatial and temporal patterns in data [33]. To achieve this, CNNs use four key features: (1) creating local connections; (2) sharing the weights; (3) a high number of layers/filters; and (4) reducing the network’s complexity [40]. As an example, in 1D CNNs, different fixed-size filters are slid over the time series. The length of the filter/window used to traverse the time series is defined as $k_{size}$. The outputs of these convolution processes between the filters and the corresponding parts of the time series are the neurons in the generated feature maps, presented in Figure 3. The associated weights of the filters and the overall bias are learned over the course of the training phase of the network and each feature map has a different set of weights. This convolution process can be formulated as [41]:

(9) $$a_{ij}^{m}=\varphi \left(b_{i}+\sum_{k=1}^{M}w_{ik}x_{j+k-1}\right)=\varphi \left(b_{i}+W_{i}^{T}X_{j}\right),$$

where $a_{ij}^{m}$ represents the output of the *j*th neuron for the *i*th filter in the *m*th convolutional layer, *φ* is the activation function, *M* is the size of the kernel, *b_i_* and $w_{i}={\left[w_{i1}\;w_{i2}\;\cdots \;w_{iM}\right]}^{T}$ are the shared bias and weights of the *i*th filter, respectively, and, finally, $x_{i}={\left[x_{j}\;x_{j+1}\;\cdots \;x_{j+M-1}\right]}^{T}$ represents an input of size *M*. As given in Equation (7) and shown in Figure 3, the same filters are applied at different locations of the input time series to generate a’s outputs. However, using different filters results in various feature maps of the input time series. The number of applied filters ($n_{filters}$) controls the size of the feature maps in all of the convolutional layers. Each filter is also defined by a set of shared weights of a size equal to *M* and a bias term. To ensure clarity, the biases for each weight in the filters are not shown in Figure 3.

### 2.7. Model Parameter Tuning

The RNN models were trained using the ADAM optimization algorithm. Unlike other well-known optimization algorithms that use a global learning rate, ADAM utilizes an adaptive learning rate to approach each parameter of the network. The proposed Stacked LSTM model was tuned for the number of LSTM layers. Various models were then constructed with an input layer followed by 1, 2, and 3 LSTM layers, respectively. The LSTM layers were followed by a dense layer with one neuron to perform single time-step forecasting. Each of the models was tuned for hyperparameters using the Keras tuner Hyperband [42]. The main hyperparameter that was tuned for was the number of units in the LSTM layers. Each of the LSTM layers was tuned separately and the search range for the number of units was from 32 to 256, with a step size of 16. The best parameters found for all three models were saved and the predictions of the three models were then extracted and compared, as described in the next section (Model Evaluation). The best-performing stacked LSTM model was compared with the CNN-LSTM and MLR models. A similar approach was taken for the CNN-LSTM model. The CNN-LSTM model was tuned for the number of 1D convolutional layers. Models were constructed with an input layer followed by 1, 2, and 3 Conv1D layers, respectively. The convolutional layers were followed by two LSTM layers and a dense layer as the output. The Conv1D layers were tuned for the number of filters in a range from 8 to 256, with a step size of 8. The three models were then compared as described in the next section (Model Evaluation). The best CNN-LSTM model was used for model comparison.

### 2.8. Model Evaluation

The REE component was added to the *VO*_2_ predictions to obtain absolute MAPE values. Each model was trained and tested 10 times to minimize the variation between each training and get a result representing the model’s ability to predict EE. The prediction from each model’s evaluation of the test set was saved to calculate the average prediction for each instance in the test set. The *VO*_2_ predictions and measurements were converted to METs by adjusting for the weight and the basal metabolic rate in the children. The Pearson correlation and RMSE were then calculated for each of the 10 subjects in the test set, by comparing the METs’ predictions from the models to the MET values measured from indirect calorimetry. A repeated measures ANOVA (RMANOVA) was used to compare the results of the models. RMANOVA was chosen since multiple samples were measured on every subject in the dataset, so the samples were not independent. RMANOVA groups samples within subjects to account for multiple samples from the same individual. The RMANOVA was conducted on the Pearson correlation and RMSE for each subject in the test set. Subsequent pairwise comparison of means was performed based on the results of the RMANOVA. The results from the RMANOVA analysis were used for selecting the optimal number of LSTM layers for the stacked LSTM model as well as the optimal number of conv1D layers in the CNN-LSTM model. The RMANOVA analyses were also used to identify the performance of the stacked LSTM and CNN-LSTM compared to the MLR baseline model.

The MAPE and correlation values for each of the nine activities in the activity protocol were calculated to assess the activity-specific weaknesses and strengths of the different model approaches. The sedentary, light, moderate, and vigorous intensity domain prediction accuracies were calculated to assess the intensity-specific measurement bias of the EE predictions models. A repeated measures two-way ANOVA and Bonferroni adjusted multiple comparison was used to evaluate if the prediction error was significantly different within the three methods and across the intensity domains. The intensity domains were determined using the thresholds 1.5, 3, and 6 METs. Correlation and MAPE were calculated in Python using functions from the Sklearn Metrics library [43]. The MAD and AGI metrics were compared by calculating the correlation and MAPE values for the test set. The calculations were performed based on the predictions from the CNN-LSTM and MLR model using each accelerometry metric.

## 3. Results

The descriptive summary statistics of the participants included in this study are presented in Table 2. There are no significant differences between genders for any of the five summary statistics.

The performance of the three EE prediction models by device placement is presented in Table 3, with the thigh sensor placement having significantly outperformed the other accelerometer placements for all models. Two-layer LSTM with CNN-LSTM, was identified as the optimal number of layers, and the result from the pairwise comparison is presented in Table A1 and Table A2, available in Appendix A. The thigh data and two-layer LSTM and CNN-LSTM were subsequently used to further evaluate the performance of the EE prediction models.

**Figure 4:**
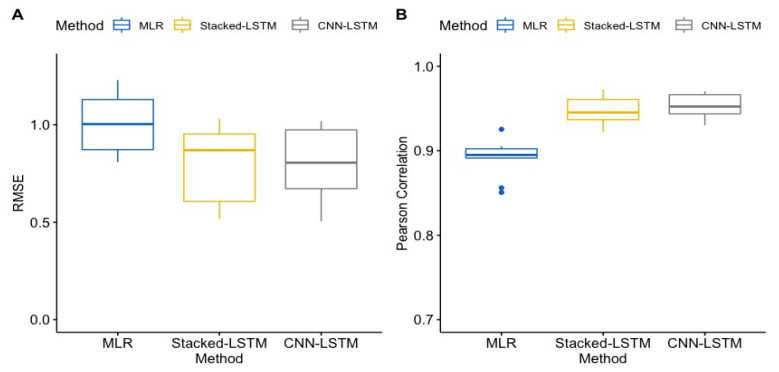
Pearson correlation (**B**) and RMSE (**A**) for predicted versus measured METs of everyone in the test set, for the three different model approaches. Data are displayed in box plots with median and inter-quartile range. Blue dots indicate outliers. MLR: multiple linear regression (baseline model). Stacked LSTM: the stacked LSTM model with two consecutive LSTM layers. CNN-LSTM: the CNN-LSTM model with two Conv1D layers.

Boxplots of the Pearson correlation and RMSE for the three EE prediction models are presented in Figure 4. The correlations ranged from 0.851 to 0.925 and the RMSE ranged from 0.808 to 1.317 for the MLR model, from 0.922 to 0.965 and 0.514 to 1.031 for the stacked LSTM, and from 0.930 to 0.970 and 0.530 to 1.019 for the CNN-LSTM model, respectively.

The results from the pairwise comparisons of the performance of the three EE prediction models are presented in Table 4. The MLR model had a significantly lower correlation than the two RNN models. There were lower correlations of 0.056 and 0.062, with *p*-values < 0.001, when the MLR model was compared to the stacked LSTM and CNN-LSTM models, respectively. No significant differences in correlation were found between the two RNN models. The CNN-LSTM seems to perform slightly better than the stacked LSTM approach, with an increase in correlation of 0.006 and a *p*-value of 0.278, though it is not significant. The comparison with the RMSE shows that the MLR model had significantly higher RMSE values, with increases in the RMSE of 0.272 and 0.277 and with *p*-values < 0.001, when compared to the stacked LSTM and CNN-LSTM, respectively. No significant differences were found between the two RNN models in terms of their RMSE. The CNN-LSTM and stacked LSTM performed nearly identically, with a small difference in their RMSEs of 0.005 and a *p*-value of 0.970. Thus, both RNN models significantly outperform the MLR model, with no significant difference between the two RNN model approaches.

Model performances for the nine activities, separately, are presented in Table 5. The two RNN models outperformed the MLR model across all activities. For the RNN models, the overall MAPE values are 14.68% and 14.22%, respectively, versus 19.89% for the MLR model.

**Figure 5:**
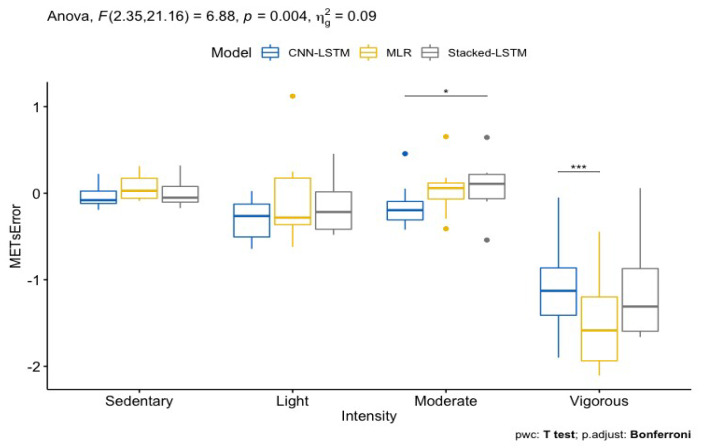
Prediction error in METs categorized in the intensity domains sedentary, light, moderate, and vigorous. Outliers are presented as dots and with * indicating *p* < 0.05 and *** *p* < 0.001.

The sedentary, light, moderate, and vigorous intensity-specific prediction errors are presented in Figure 5. The prediction error for vigorous intensities is significantly different (*p* < 0.01) from all of the other intensities and that for moderate intensities is significantly different with the stacked LSTM as compared to the CNN-LSTM. For vigorous intensities, the CNN-LSTM is significantly different from the MLR. A repeated measures Bland Altman analysis was used to evaluate the intensity-specific biases and limits of agreement (LOA). The estimated bias for sedentary, light, and moderate intensities ranges from −0.3 to 0.08 METs, with a lower LOA from −1.74 to −0.63 METs and an upper LOA from 0.57 to 1.81 METs, whereas, for vigorous intensities, the bias ranges from −1.5 to −1.08 METs, with a lower LOA from −3.95 to −3.01 METs and an upper LOA from 0.86 to 0.95 METs. The individual intensity-specific biases and upper and lower LOAs determined with the Bland Altman analysis are presented in Table A3 in Appendix A.

The performance of the MLR and CNN-LSTM models for the MAD and AGI PA metrics, separately, is presented in Table 6. The R^2^ of the MLR model is lower for MAD as compared to AGI, with a difference in MAPE of 5,64%. The correlation of the CNN-LSTM model is also in favor of AGI as compared to MAD, with a decrease in the MAPE of 1.04%, from 14.22% for the AGI model to 15.26% with the MAD model. The differences in the correlation and MAPE are smaller for the CNN-LSTM models as compared to the MLR.

## 4. Discussion

In this study, we investigated EE prediction from acceleration and the importance of incorporating the temporal elements of movement by using LSTM and CNN-LSTM recurrent neural networks. Both stacked LSTM and CNN-LSTM networks significantly outperformed the reference MLR model on all performance metrics, suggesting that including the temporal elements of movement is important to obtain an accurate EE prediction from acceleration.

Predicting EE from acceleration measured at the thigh, as investigated in the present study, has only been addressed in one previous study, conducted by Montoye et al. [44]. In their study, the EE prediction was analyzed using linear regression, mixed linear regression, and neural networks. For the linear regression model, the findings of the Pearson’s correlation and RMSE are comparable between the two studies. However, the LSTM models in this study outperformed the results of the proposed neural networks by Montoye et al. [44]. Our proposed LSTM models perform in the range of 0.922–0.970 and 0.53–1.03 in terms of R^2^, compared to the ranges of only 0.71–0.88 and 1.11-1-61 presented in Montoye et al. [44]. This may be due to the differences in population, epoch length, and activities included in the protocol of the two studies. The epoch length and population addressed in this study are 10 s and children, as compared to 30 s and adults in Montoye et al. [44]. However, when using a shorter epoch length and child population, it is expected to be more challenging to predict EE accurately due to the intermittent and sporadic nature of their movements. Moreover, in this study, we included an intermittent activity, which is not considered in Montoye et al. [44].

To investigate the validity and generalizability of the proposed models with actual collected data in a natural environment, we evaluated the prediction error across the intensity domains sedentary, light, moderate, and vigorous but also for the nine activities in the protocol separately. The results clearly show that the EE prediction error for vigorous intensities is significantly different as compared to all other intensity domains. Moreover, the variation in the prediction error across the different intensity domains also seems to indicate some heteroscedasticity. The overall picture seems to indicate a systematic bias, especially with high intensity activities, and suggests that the models do not provide a perfect external validity. There might be several reasons for this, but our primary suggestion is differences in movement efficiency. During the execution of the activities, we observed a large variation in movement efficiency, with several children having difficulty sustaining running at a low speed for 5 min while others could maintain 10–14 km/h for the entire duration. Thus, acceleration and locomotion speed alone are most likely not sufficient to account for the huge differences in movement efficiency. Further research seems to be required to investigate EE prediction with vigorous activities in relation to movement efficiency.

The basketball and sitting with a tablet activities had negative R^2^ values, which is counterintuitive. A negative R^2^ value is caused by the data set being imbalanced regarding the number of data samples in the different activities. Over 50% of the data samples in the test set are labeled “break between activities”, and this will account for most of the influence on the total R^2^ value, which is also why the R^2^ value of all the activities combined closely resembles the R^2^ for the break activity. However, negative R^2^ values for a single activity do not affect the R^2^ value of the totality of the test set.

The prediction accuracies for both RNN approaches are quite similar, meaning that the decrease in prediction error when comparing RNN models to a MLR model might be attributed to the fact that LSTM layers account for temporal dependencies in EE prediction. However, this could also stem from using a nonlinear approach. For future work, it could be interesting to examine the efficiency of other machine learning algorithms such as Random Forest or SVM models and compare them to RNN models. Other studies have conducted EE prediction from accelerometry using machine learning [8,9,10,11,12,13,14,15,16,17,18,19,20], but the results are hard to compare since the demography, features, and sensor placements are not similar.

There are numerous methods available for generating intensity-specific metrics from acceleration and, in this study, we investigated the effect of using AGI compared to MAD on the prediction accuracy. We expected to observe an improved prediction accuracy with the AGI as compared to MAD, as AGI uses an enveloping of high-intensity activity to account for the elevated level of PA EE caused by EPOC. However, there is no apparent increase in accuracy, at least for the CNN-LSTM model, which might suggest that the CNN-LSTM model is able to generalize from the pattern that the rapid component of EPOC causes a delayed lowering of EE after time periods of high intensity. Comparing the results of the CNN-LSTM and the MLR model, it seems that using AGI is more effective than MAD using the MLR model but not using CNN-LSTM. This supports the expectation that AGI would have a positive effect on the performance of conventional model types when predicting EE. The fact that the CNN-LSTM model can learn temporal patterns makes RNN models a much more appealing approach since accelerometer metrics such as AGI require complex data preprocessing steps before the data are ready for the model to predict. With RNN models, these preprocessing steps can be omitted.

## 5. Conclusions

In this study, we evaluated EE prediction from acceleration measured at the thigh, hip, wrist, and back of children, using different machine and deep learning models, and compared them to standard linear regression. The findings from this study demonstrate that the thigh provides an improved prediction accuracy as compared to the other placements but also, importantly, that utilizing the temporal aspects of movement improves the accuracy of EE prediction. The prediction accuracy was evaluated with two different PA metrics generated from acceleration, and there was only a limited increase in accuracy when accounting for the sporadic and intermittent nature of children’s PA behavior within the accelerometry feature rather than using the models developed to predict EE. The measurement bias observed with vigorous intensity activities and basketball seems to suggest that further investigation is required.

## Figures and Tables

**Table 1:** The activities performed by the participants during the structured protocol [28]. Playground activity was only performed by nine participants. During the transition between the planned activities the subjects were either sitting, standing, or walking.

Order	Activity	Description
1	Sitting	Sitting on a chair close to a table with arms in the lap
2	Sitting playing	Playing the Fruit Ninja game on an iPad
3	Standing playing	Playing a game on the iPad while standing
4	Slow walking	Slow walking speed
5	Brisk walking	Brisk walking speed
6	Running	Running at the subjects own preferred running speed
7	Basketball	One-to-one competitive basketball game play
8	Playground	Running and walking around the school playground
9	Biking	Commuting cycling on subjects’ own bike
10	Sitting	Sitting close to a table with arms in the lap

**Table 2:** Descriptive statistics of the participants included in the dataset with valid accelerometer data. The mean ± standard deviation is presented with the minimum and maximum values inside the parentheses.

	Boys (n = 18)	Girls (n = 15)	All (n = 33)
Age (years)	10.5 ± 0.7 (9.4, 11.6)	10.5 ± 0.7 (9.3, 11,8)	10.5 ± 0.7 (9.3, 11.8)
Weight (kg)	39.4 ± 7.9 (26.2, 53.0)	38.8 ± 5.5 (30.8, 51)	39.0 ± 6.8 (26.2, 53.0)
Height (cm)	145.7 ± 7.6 (132.2, 159.5)	145.1 ± 6.6 (134.5, 158.5)	145.5 ± 7.2 (132.2, 159.5)
BMI (kg/m^2^)	18.4 ± 2.4 (15.0, 23.0)	18.3 ± 2.1 (14.5, 22.0)	18.3 ± 6.8 (14.5, 23.0)
REE (ml/kg/min)	4.7 ± 0.9 (3.1, 6.3)	4.6 ± 0.7 (3.7, 6.1)	4.6 ± 0.81 (3.1, 6.3)

**Table 3:** R^2^ and MAPE for the four different device placements. Bold text indicates the best-performing placement of the sensor for each metric within each model.

Model Architecture:	MLR		Stacked LSTM		CNN-LSTM	
Sensor Placement	R^2^	MAPE (%)	R^2^	MAPE (%)	R^2^	MAPE (%)
Back	0.564	33.6%	0.702	29.5%	0.772	22.0%
Wrist	0.485	33.1%	0.745	19.1%	0.776	18.1%
**Thigh**	**0** **.** **76**	**19.** **9** **%**	**0** **.** **872**	**14.9%**	**0** **.** **883**	**13.** **9** **%**
Hip	0.562	32.6%	0.757	24.7%	0.795	18.9%

**Table 4:** Results of the pairwise comparisons of model performance based on RMANOVA.

Metric	Model 1	Model 2	Mean Difference	Standard Deviation	*p*-Value
RMSE	MLR	Stacked LSTM	0.272	0.029	<0.001
RMSE	MLR	CNN-LSTM	0.277	0.026	<0.001
RMSE	Stacked LSTM	CNN-LSTM	0.005	0.020	0.970
Correlation	MLR	Stacked LSTM	−0.056	0.008	<0.001
Correlation	MLR	CNN-LSTM	−0.062	0.006	<0.001
Correlation	Stacked LSTM	CNN-LSTM	−0.006	0.004	0.278

**Table 5:** Prediction error for activities, separately.

Model Architecture	MLR	Stacked LSTM	CNN-LSTM
Metric	MAPE (%)	MAPE (%)	MAPE (%)
Sitting	12.82	9.87	12.27
Sitting w. tablet	23.91	20.28	19.61
Standing w. tablet	30.28	16.84	10.19
Walking (pref. speed)	11.37	9.52	10.06
Walking (brisk)	7.31	9.71	9.62
Running	15.97	12.32	11.50
Basketball	31.09	18.39	19.19
Biking	20.68	19.55	19.30
Break	20.83	14.70	14.37
All activities	19.89	14.68	14.22

**Table 6:** The performance of the CNN-LSTM model and the linear regression model for MAD and AGI.

	R^2^		MAPE	
Model	MAD	AGI	MAD	AGI
Multiple Linear Regression	0.677	0.778	25.5%	19.9%
CNN-LSTM	0.847	0.879	15.3%	14.2%

## Data Availability

The dataset generated and/or analyzed during the current study are not publicly available due to GDPR rules but are available from the corresponding author on reasonable request.

## Appendix A

Table A1 presents the RMANOVA analysis and subsequent comparisons of means conducted to identify the optimal number of LSTM layers in the stacked LSTM model. There is no significant difference between the models with 1 LSTM layer and the model with three consecutive LSTM layers. This is the case for both RMSE and correlation. The stacked LSTM model with 2 LSTM layers outperforms the other models with a RMSE value that is significantly lower, with a *p*-value of 0.036 and 0.037 for the one-LSTM-layer and three-LSTM-layer models, respectively. The same can be seen for correlation, with a significantly higher correlation value with a *p*-value of 0.038 and 0.024 compared to the one-LSTM-layer and three-LSTM-layer models, respectively. The 2-layer model was chosen to represent the stacked LSTM model approach.

**Table A1:** Results of RMANOVA and comparison of means on the predictions from the stacked LSTM models containing 1 LSTM layer, 2 consecutive LSTM layers, and 3 consecutive LSTM layers. Model 1 and Model 2 describe the pair of models which are compared. The difference of means takes reference in the mean of Model 1.

Metric	Model 1	Model 2	Mean Difference	Standard Deviation	*p*-Value
RMSE	1 layer	2 layers	0.073	0.024	0.036
RMSE	1 layer	3 layers	0.003	0.023	0.988
RMSE	2 layers	3 layers	−0.070	0.023	0.037
Correlation	1 layer	2 layers	−0.015	0.005	0.038
Correlation	1 layer	3 layers	−0.001	0.004	0.934
Correlation	2 layers	3 layers	0.014	0.004	0.024

Table A2 presents the RMANOVA analysis and subsequent comparisons of means conducted when tuning for the number of Conv1D layers in the CNN-LSTM model. There is no significant difference between any of the models, neither in correlation nor RMSE. The model with 2 Conv1D layers does seem to outperform the other models slightly, with a slight difference in RMSE values of −0.009 and −0.006 and *p*-values of 0.861 and 0.952, when compared to the 1-layer and 3-layer models, respectively. The same is the case for correlation, with a slight difference in correlation of 0.002 and 0.003 and *p*-values of 0.612 and 0.406, when compared to the 1-layer and 3-layer models, respectively. The model with 2 layers was chosen as the best model, since it performs slightly better than the other two, even if the difference is not significant.

**Table A2:** Results of RMANOVA and comparison of means on the predictions from the CNN-LSTM models containing 1 Conv1D layer, 2 Conv1D layers, and 3 consecutive Conv1D layers. Model 1 and Model 2 describe the pair of models which are compared. The difference in means takes reference in the mean of Model 1.

Metric	Model 1	Model 2	Mean Difference	Standard Deviation	*p*-Value
RMSE	1 layer	2 layers	0.009	0.016	0.861
RMSE	1 layer	3 layers	0.002	0.016	0.987
RMSE	2 layers	3 layers	−0.006	0.021	0.952
Correlation	1 layer	2 layers	−0.002	0.002	0.612
Correlation	1 layer	3 layers	0.003	0.002	0.406
Correlation	2 layers	3 layers	0.003	0.002	0.406

**Table A3:** Sedentary, light, moderate and vigorous intensity specific Bland Altman analysis.

Model	Sedentary	Light	Moderate	Vigorous
MLR	0.06 (−0.77, 0.89)	−0.07 (−1.74, 1.61)	0.03 (−1.74, 1.81)	−1.50 (−3.95, 0.95)
Stacked-LSTM	0.00 (−0.63, 0.63)	−0.16 (−1.32, 1.01)	0.08 (−1.57, 1.74)	−1.14 (−3.17, 0.89)
CNN-LSTM	−0.04 (−0.64, 0.57)	−0,30 (−1.22, 0.61)	−0.14 (−1.54, 1.25)	−1.08 (−3.01, 0.86)
